# Optical Access to the Electronic Nature of Device-Relevant Dislocations in High-Purity Semi-Insulating SiC

**DOI:** 10.1021/acsomega.6c02027

**Published:** 2026-06-25

**Authors:** Hsiu-Ming Hsu, Irwan Saleh Kurniawan, Russel Cruz Sevilla, Ruth Jeane Soebroto, Sheng-Hsiung Chang, Troy Tsai, Hsiu-Ying Huang, Wen-Chung Li, Chi-Tsu Yuan

**Affiliations:** † Department of Physics, 34900 Chung Yuan Christian University, 320314 Taoyuan, Taiwan; ‡ Research Center for Semiconductor Materials and Advanced Optics, Chung Yuan Christian University, 320314 Taoyuan, Taiwan; § Department of Optics and Photonics, 34911 National Central University, 320317 Taoyuan, Taiwan; ∥ LEAP Semiconductor Corporation, 330007 Taoyuan, Taiwan; ⊥ Wafer Works, 325002 Taoyuan, Taiwan

## Abstract

High-purity semi-insulating silicon carbide (HPSI-SiC) is a high-end substrate for power devices. However, both electrical and optical characterization of threading dislocations (TDs) remains challenging because of its low free-carrier concentration and abundant compensating deep-level defects. Here, we develop an optical technique for the selective identification and electronic characterization of device-relevant TDs with continuously distributed deep-level states. By employing partially etched TDs, TD types can be resolved through laser backscattering from etch-pit morphology, while their electronic activity is assessed via deep-level photoluminescence (PL) from the underlying dislocation lines. Statistical one-to-one structural–electronic correlation reveals that only a small fraction of pure screw-type TDs exhibits broadband deep-level emission, which is attributed to inherent dislocation-core states. These deep-level TDs may potentially form leakage-current pathways through trap-assisted mechanisms. Our work demonstrates a nondestructive PL-active approach for optically identifying deep-level TDs in HPSI-SiC.

## Graphical abstract

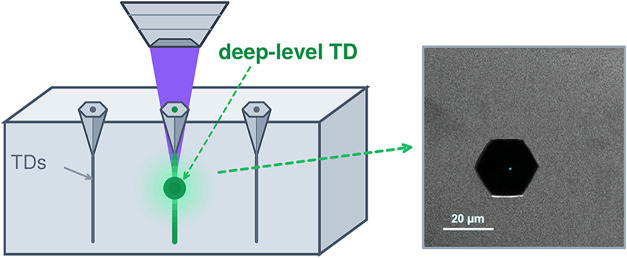

## 1 Introduction

High-purity semi-insulating silicon carbide (HPSI-SiC) features a wide bandgap, high thermal conductivity, low intrinsic carrier density, and excellent electrical isolation, making it a high-end substrate for GaN-based RF amplifiers and high-voltage power switches.^1–3^ However, despite these advantageous properties, HPSI-SiC still contains a high density of threading dislocations (TDs), a small subset of which can critically affect device performance and reliability via their deep-level states, referred to as deep-level TDs (DL-TDs).^4,5^ These electrically active DL-TDs may propagate into the epilayers, acting as the leakage current paths and carrier trapping sites, thereby compromising the semi-insulating nature and degrading the device operation.^6,7^

Effective defect inspection and characterization in HPSI-SiC, particularly for device-relevant DL-TDs, is critical for ensuring substrate quality prior to GaN epitaxial growth.^8,9^ Electrically active DL-TDs present in the HPSI-SiC substrate may influence the defect formation in the subsequently grown GaN epilayers and further extend into the active region of the device, creating the leakage paths or parasitic conduction channels.^10,11^ Moreover, the termination points of DL-TDs may act as nucleation sites for other extended defects.^12^ From this perspective, the ability to identify such defects in the substrate prior to GaN epitaxial growth is highly desirable. However, due to the semi-insulating nature and low intrinsic carrier density, conventional electrical characterization techniques such as deep-level transient spectroscopy (DLTS) and electron-beam induced current (EBIC) are often limited in applicability.^13,14^ To date, only a few nonelectrical approaches, such as defect-selective etching and X-ray topography have been employed to visualize and analyze the TDs.^15–17^ Nevertheless, these techniques respond indiscriminately to all TDs and lack the ability to selectively detect and identify device-relevant DL-TDs that would critically impact device performance.

It should be noted that despite the high TD density in SiC (on the order of 10^3^ to 10^4^ cm^–2^), only a small subset possesses deep-level states and could be electrically active for device operation. These DL-TDs could form conduction pathways via continuously distributed deep-level states based on defect-assisted tunneling or Poole-Frankel emission models.^18–21^ Unfortunately, conventional strain-based techniques, such as X-ray topography (XRT) and defect-selective etching, which rely on structural distortions, are unable to selectively identify DL-TDs.^22^

Photoluminescence (PL)-based techniques are widely employed in n-type SiC epilayers with lower doping concentration and have become industry-standard methods for in-line defect inspection, typically relying on defect-induced emission quenching of band-edge PL.^23–25^ However, in HPSI-SiC substrate, these techniques become largely ineffective due to the presence of abundant background deep-level defects (e.g., *Z*_1/2_ centers).^26^ These defects can efficiently trap the free electrons to compensate residual donors and also act as carrier lifetime killers, suppressing radiative recombination and significantly reducing the PL intensity. In this case, the photogenerated carriers predominantly recombine nonradiatively through background deep-level defects and DL-TDs, resulting in significant quenching of the band-edge emission and consequently reduced imaging contrast.^23,26,27^

To date, PL-based techniques for optical characterization in HPSI-SiC have been rarely reported, and no studies have specifically targeted TDs, particularly those that are electrically active and critical to device reliability and leakage behavior. This reveals a significant gap in the optical identification and electronic assessment of device-relevant DL-TDs in HPSI-SiC substrates. While only a few investigations have observed NIR emissions associated with point defect–related color centers.^28,29^

To address these challenges, we exploit deep-level-state-mediated emission to identify device-relevant DL-TDs in HPSI-SiC substrates. This approach enables selective probing of DL-TDs even in the presence of abundant compensating *Z*_1/2_ defects, while providing direct insight into their electronic properties. By correlating dislocation etch-pit morphology with deep-level emission from the corresponding dislocation lines, we resolve the nature of device-relevant DL-TDs. Remarkably, we find that only a small subset of pure TSDs exhibits continuously distributed deep-level states, making them potential leakage-current pathways. These states mainly originate from the dislocation-core structure, highlighting the potential of our approach as a powerful nondestructive inspection method for DL-TDs.

## 2 Experimental Methods

### 2.1 Materials and Sample Preparation

A 4-in. high-purity semi-insulating (HPSI) 4H-SiC wafer (thickness: 500 μm, 0° on-axis orientation) was obtained from SICC Co., Ltd. The wafer was grown by physical vapor transport (PVT) using high-purity source powders, cut along the *c*-axis, and chemo-mechanically polished to provide an epi-ready surface with low roughness and high flatness. For experiments, the wafer was cleaved into ∼0.2 × 0.5 cm^2^ samples to facilitate microscope handling. To reveal crystallographic defects, the samples were etched in molten KOH at 510 °C for 15 min, producing well-defined etch pits that served as surface markers for subsequent confocal laser scanning spectro-microscopy.

### 2.2 Confocal Dual-Mode Spectro-Microscopy

Optical measurements were performed using a confocal microscope (PicoQuant MicroTime 100) integrated with a spectrometer for combined imaging and spectral acquisition. Excitation was provided by a 375 nm pulsed diode laser, coupled into a single-mode optical fiber, and focused onto the sample using an oil-immersion objective with a numerical aperture of 1.4. Emission signals were detected by two single-photon avalanche diodes (SPADs) operated in parallel. A 380 nm band-pass filter was used to collect laser backscattering signals, while a 400 nm long-pass filter was used to collect defect-related PL, enabling simultaneous laser backscattering and defect-PL imaging. For spectral measurements, the signal was fiber-coupled into a monochromator and detected using a cooled photomultiplier tube. A three-dimensional piezoelectric scanner mounted on the objective was used for lateral and cross-sectional mapping.

## 3 Results and Discussion

### 3.1 Revelation of Dislocation Etch Pits in HPSI-SiC

Dislocation etch pits (DEPs) were revealed in HPSI-SiC through defect-selective etching with molten KOH, enabling both the localization of TDs and the identification of their dislocation types based on the etch pit morphology. Notably, once the optical signatures of DL-TDs are established in this work, such pre-etching steps become unnecessary. As shown in Figure 1(a,b), a wide-area SEM image reveals the DEPs with varied shapes, sizes, and patterns. It should be noted that the DEP morphology can be determined by both vertical and lateral etching rates, which are influenced by several factors, such as dislocation types (Burgers vector), core structure, impurity/point defect decoration, local strain fields, and etching conditions.^30^

**Figure 1:**
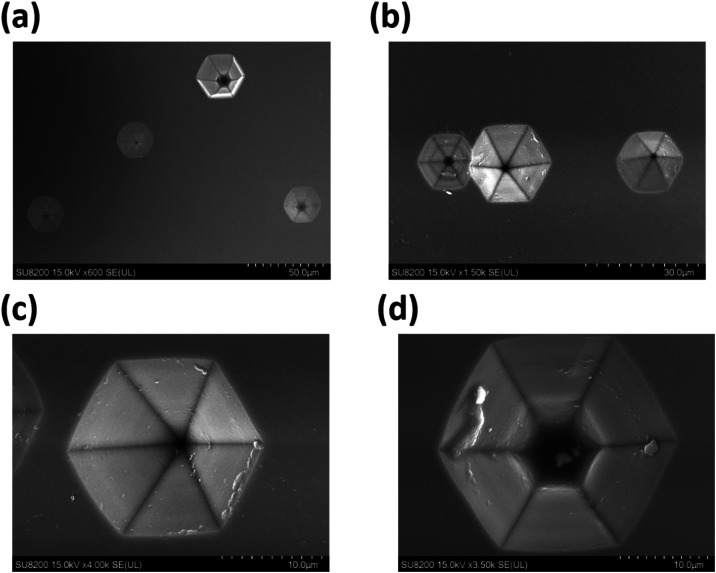
SEM images of dislocation etch pits (DEPs) in HPSI-SiC substrates. (a, b) Wide-area view showing multiple DEPs with varied sizes and morphologies. (c) Most prevalent DEP type exhibiting single hexagonal patterns with asymmetric six facets, assigning to the TSDs. (d) Representative example of double hexagonal DEP morphology, assigning to the TMDs.

Here, we focus exclusively on screw-type TDs, including pure threading screw dislocations (TSDs) and threading mixed dislocations (TMDs), due to their significant impact on device performance.^31^ However, these two types of TDs often exhibit similar DEP dimensions, making it difficult to distinguish them based solely on size, a method commonly used previously. Therefore, more detailed differentiation criteria, such as etch pit depth profiles and morphological pattern characteristics, are necessary to accurately classify these dislocations and to further analyze their structural features and associated electronic behaviors.

Figure 1(c,d) show representative SEM images of specific DEPs we are interested here. The DEP shown in Figure 1(c) corresponds to the more commonly observed patterns (85% occurrence), featuring a single hexagonal structure with noticeable asymmetry. This characteristic morphology is attributed to the TSD with only *c*-axis Burgers vector, while the observed asymmetry suggests that local etching behavior is influenced by nearby compensating defects. In contrast, Figure 1(d) displays a more complex DEP with a distorted double-hexagonal structure, resulting from combined lateral and vertical etching dynamics. This pattern is assigned to the TMD, possessing both *a*-axis and *c*-axis components in its Burgers vector.^15^

### 3.2 Identification the TD Types

To confirm the assignment of the TD types, we also performed confocal laser backscattering mapping on both lateral and cross-sectional planes, enabling reliable identification of DEPs via surface patterns and depth profiles. This method not only provides valuable insight into the DEP morphology but also facilitates one-to-one structural–electronic correlation for individual TDs with a partially etched configuration (Supporting Information, Figure S1).

Figure 2 presents surface and depth-profile confocal laser backscattering mappings of two representative TDs. The surface image (Figure 2(a)) shows a well-defined hexagonal etch pit with a dark region and six facet boundary lines, while the corresponding cross-sectional view (Figure 2(b)) reveals a deep triangular profile, characteristic of the TSDs. In contrast, Figure 2(c,d) show the surface and depth profiles of another type of TD, distinguished by double-hexagonal etch pits arising from two slope depths with six-faceted sidewalls, together with a curved subsurface profile. These morphological features are characteristic of the TMD, which contains both *a*-axis and *c*-axis Burger’s vector components.

**Figure 2:**
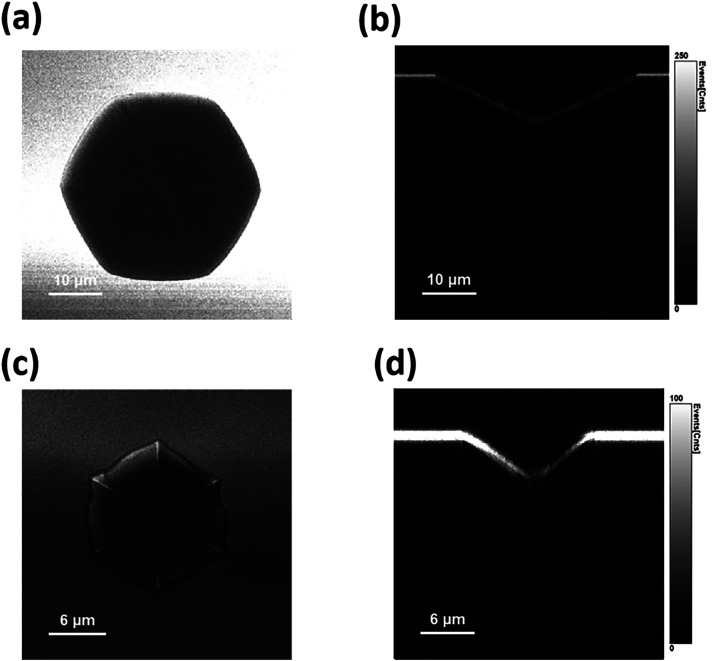
Confocal laser backscattering mappings of two representative TDs. (a, b) Surface and cross-sectional views of an etch pit associated with the TSD with a hexagonal pattern and a triangular depth profile. (c, d) Corresponding surface and depth-profile images of the TMD, characterized by a double hexagonal pattern and a curved depth profile.

### 3.3 Deep-Level defect-PL Mapping of Dislocation Lines

While laser backscattering imaging provides valuable information on the DEP morphology, it does not yield direct insight into the electronic activity of the TDs, which is more critical for device performance. PL imaging is widely used to study extended defects; however, conventional approaches, relying on band-edge emission quenching at defect sites, are ineffective for TDs in HPSI-SiC substrates. This ineffectiveness arises from the high density of compensating *Z*_1/2_ defects and the intrinsically low free-carrier concentration, both of which diminish imaging contrast. To overcome these limitations, we employ a defect-mediated PL technique that directly probe the deep-level emissions from the dislocation lines (hereafter referred to as defect-PL).^32,33^ This method enables direct study of the electronic activity of device-relevant DL-TDs, bypassing the constraints of band-edge PL-quenching techniques.

Figure 3 shows a representative image set of a typical TSD, whose DEP morphology was identified using both surface and cross-sectional laser backscattering mapping (Figure 3(a,b)). In the cross-sectional defect-PL image (Figure 3(c)), the TSD exhibits a bright etch pit profile and a nonemissive dislocation line (thus not observable). It should be noted that the bright emission from the etch pit is attributed to radiative recombination via surface states exposed at hexagonal facets of the etch pits. Notably, no emission is detected along the dislocation lines, as further confirmed by subsurface defect-PL mapping (Figure 3(d)), indicating the absence of optically active deep-level states in this TSD. Such electronically inactive TSDs constitute the majority of TDs in our sample. A similar behavior was also observed for TMDs, which display bright etch pits but dark dislocation lines (Supporting Information, Figure S2).

**Figure 3:**
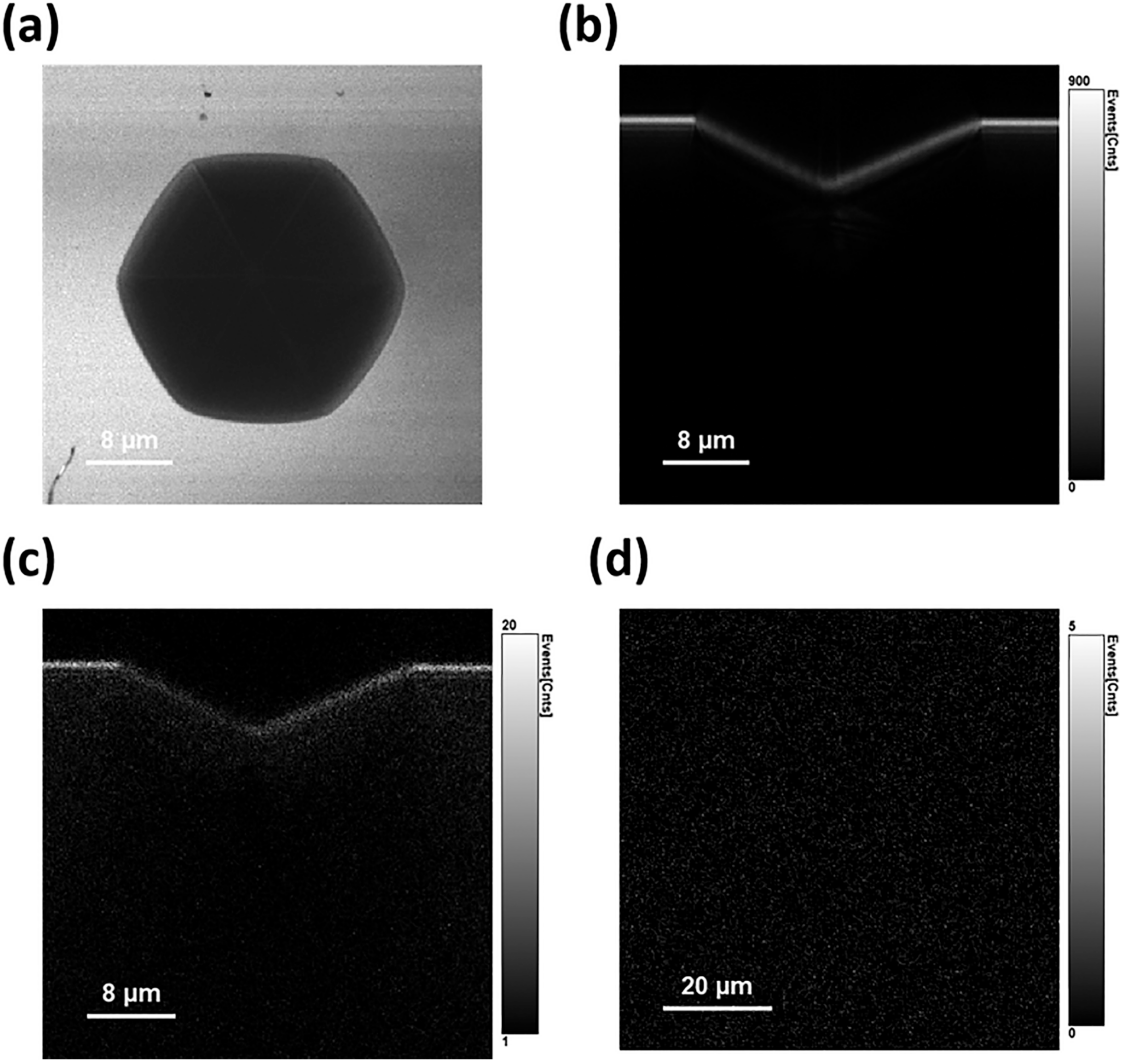
Correlated laser backscattering and defect-PL images of a specific TSD. (a) Surface laser backscattering mapping of etch pits. (b) Cross-sectional laser backscattering mapping of the same etch pits. (c) Cross-sectional defect-PL mapping of the etch pits. (d) Subsurface defect-PL mapping of dislocation lines beneath the etch pits, implying no optically active deep-level states.

Remarkably, a very small fraction of the TSDs (∼1%) exhibited distinct behavior, as shown in Figure 4. Despite having similar DEP morphology (Figure 4(a)), such specific TSDs displayed both bright etch pits and bright dislocation lines in the depth-profile defect-PL images (Figure 4(b)). To confirm the emissions arising from the dislocation lines, we also performed subsurface defect-PL mapping at a few microns beneath the DEP (Figure 4(c)). Indeed, a distinct bright spot was clearly observed at the dislocation core location, indicating that the emission originates from the dislocation cores. Furthermore, the overlay of surface laser backscattering and subsurface defect-PL images (Figure 4(d)) confirms the spatial correlation between the dislocation line and the localized emission. These experimental results provide robust evidence that the observed emission arises from the dislocation lines of specific DL-TDs, rather than from the surface states of the DEPs or background impurity.

**Figure 4:**
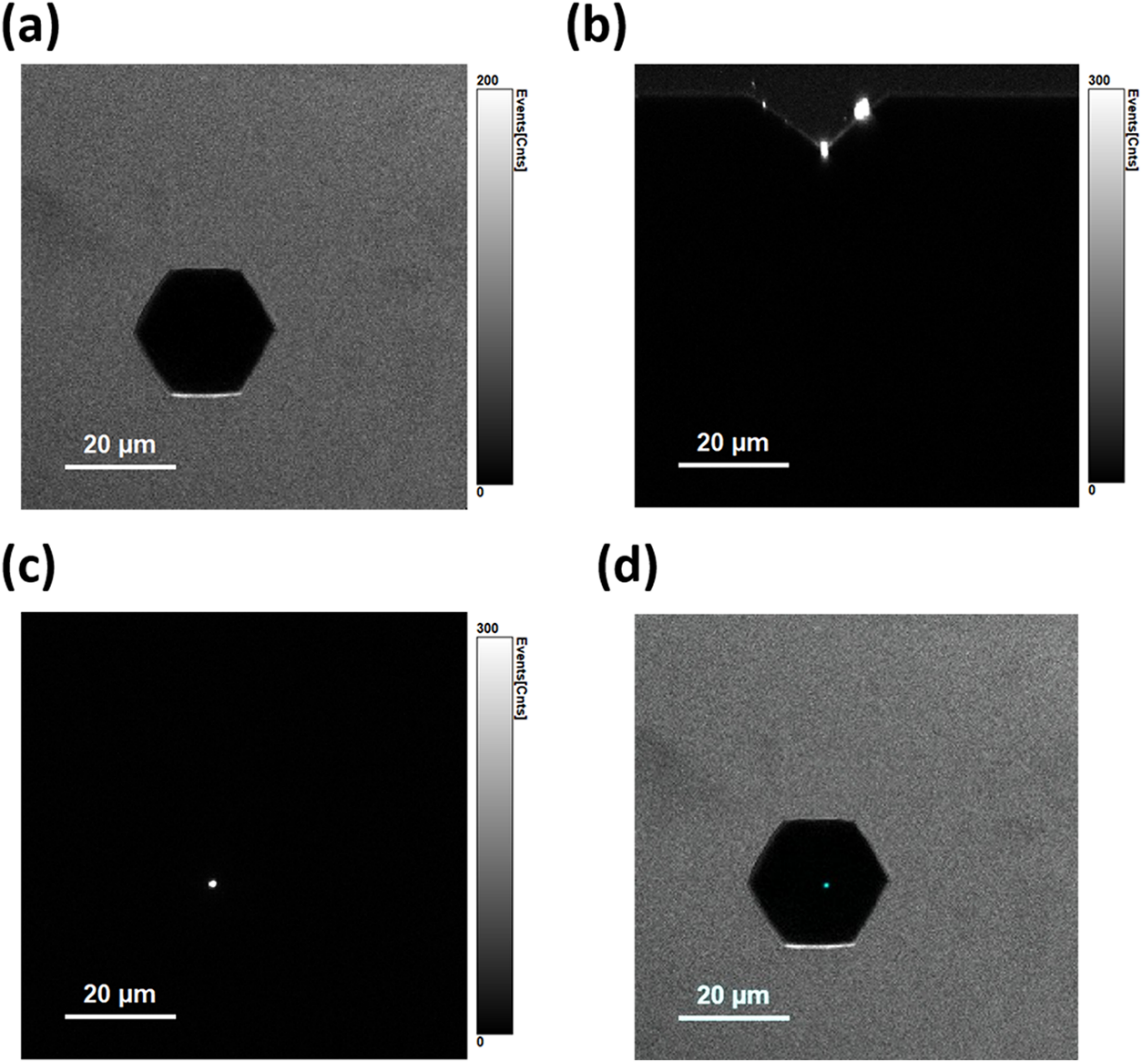
One-to-one structural and electronic correlation for a specific TSD with deep-level state emissions. (a) Surface laser backscattering mapping of the etch pits. (b) Cross-sectional defect-PL mapping of the entire TSDs, including etch pits and dislocation lines. (c) Subsurface defect-PL mapping of the dislocation lines. (d) Overlay of surface laser backscattering and subsurface defect-PL images, implying the deep-level defect emissions of the dislocation lines.

Figure 5 presents a set of corresponding images for two nearby TDs, enabling direct comparison. Surface laser backscattering mapping (Figure 5(a)) was employed to determine the dislocation types, identifying both defects as TSDs despite their different sizes, consistent with SEM observations (Figure 1(b)). Subsurface defect-PL mapping (Figure 5(b)), together with overlaid images combining surface backscattering and defect-PL signals (Figure 5(c)), clearly reveals that only the TSD on the right side exhibits a bright localized emission spot. This contrast underscores the distinct electronic activities of structurally similar TSDs and highlights the ability of our technique to selectively and nondestructively identify deep-level DL-TDs, those most likely to be device-relevant through their deep-level states.

**Figure 5:**
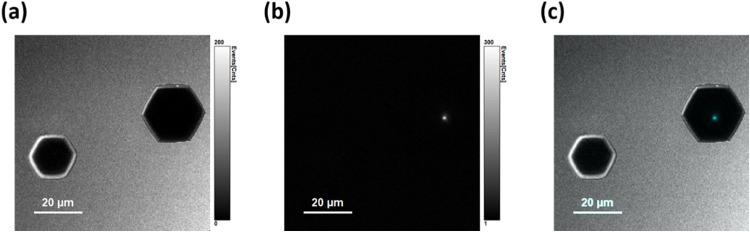
One-to-one structural and electronic correlation for two adjacent TDs. (a) Surface laser backscattering mappings. (b) Subsurface defect-PL mapping, only revealing a single bright spot attributed to bright deep-level state emissions. (c) Overlay image combining surface backscattering and subsurface defect-PL images, showing the spatial coincidence of the bright PL spot with the etch pits on the right side.

Based on a statistical one-to-one analysis of over 1000 TDs, we found that only a small fraction of TSDs (approximately 1%) exhibited bright deep-level emissions of the dislocation lines. This result is consistent with previous observations that only a limited subset of TDs is detrimental to the device performance.^34,35^ Given the extremely low dopant concentration in HPSI-SiC, these emissions can be attributed to intrinsic deep-level states associated with the dislocation cores, such as dangling bonds and wrong bonds, rather than impurity decoration.^36^ This indicates that certain TSDs inherently host abundant deep-level states, which can be optically activated. As these deep levels can mediate charge trapping and detrapping, they could also facilitate field-induced emission or carrier hopping under high electric fields. Consequently, such TSDs are likely to be electrically active as well, potentially contributing to leakage currents in related devices.^37,38^

### 3.4 Unraveling Electronic Properties of Inherent Deep-Level States

Conventional PL-based techniques, which rely on defect-induced quenching of band-edge emission, provide only limited insight into defect electronic properties. In contrast, our PL-active mode technique captures radiative emissions mediated by deep-level states of the defects, enabling direct probing of their electronic states for device-relevant TDs.^39^ While dislocation-related emissions have been documented in silicon and diamond,^40,41^ their observation in HPSI-SiC is fundamentally constrained by the material’s semi-insulating nature. In this context, the work by Nagano et al. regarding low-doped (∼10^14^ cm^–3^) 4H-SiC epilayers provides a critical physical benchmark.^42^ In their study, the low background doping preserves long carrier lifetimes, allowing photoexcited carriers to diffuse into deep regions (∼120 μm) and subsequently undergo radiative recombination at TD sites. This results in bright-spot PL signatures in the near-infrared (NIR) region (600–1000 nm), with specific peaks reported for TSDs (800–950 nm) and TEDs (>950 nm). Nagano et al. attribute these NIR signatures to trap states formed by bond reconstruction such as Si–Si or C–C bonds along the dislocation cores.

However, in HPSI-SiC, physics changes significantly. The high density of intentional compensating deep acceptors drastically reduces carrier lifetime and quenches the carrier diffusion required for Nagano’s mechanism. Our approach overcomes this by utilizing direct photoionization of occupied deep-level states within the TD cores. The resulting emission is centered in the visible range at ∼2.5 eV a significant blue-shift compared to the NIR peaks (1.3–1.5 eV) seen in epilayers. While the NIR signatures in epilayers likely originate from shallow-to-midgap traps associated with core reconstruction, the visible spectra observed here are likely associated with higher-energy transitions between intrinsic core states, such as dangling bonds, wrong bonds or severely distorted lattice configurations.

The defect-PL spectra of two independent optically active TSDs are shown in Figure 6(a), both spectra exhibiting nearly identical broadband emission profiles in the visible range, with a peak centered at ∼2.5 eV. In general, three types of electronic transitions can be associated with deep-level states: (i) defect-bound excitonic emission, (ii) free-to-bound emission, and (iii) donor-to-acceptor pair (DAP) emission.^43,44^ Dislocation-bound excitonic emission is expected to exhibit a narrower line width characteristic of bound excitons; the observed broadband spectrum is therefore inconsistent with this mechanism and can be ruled out. Impurity-related DAP recombination is also unlikely due to very low impurity concentration in our sample. This assessment is corroborated by the absorption spectrum (Figure 6(b)), which shows pronounced absorption only in the UV region, with negligible absorption in the visible.

**Figure 6:**
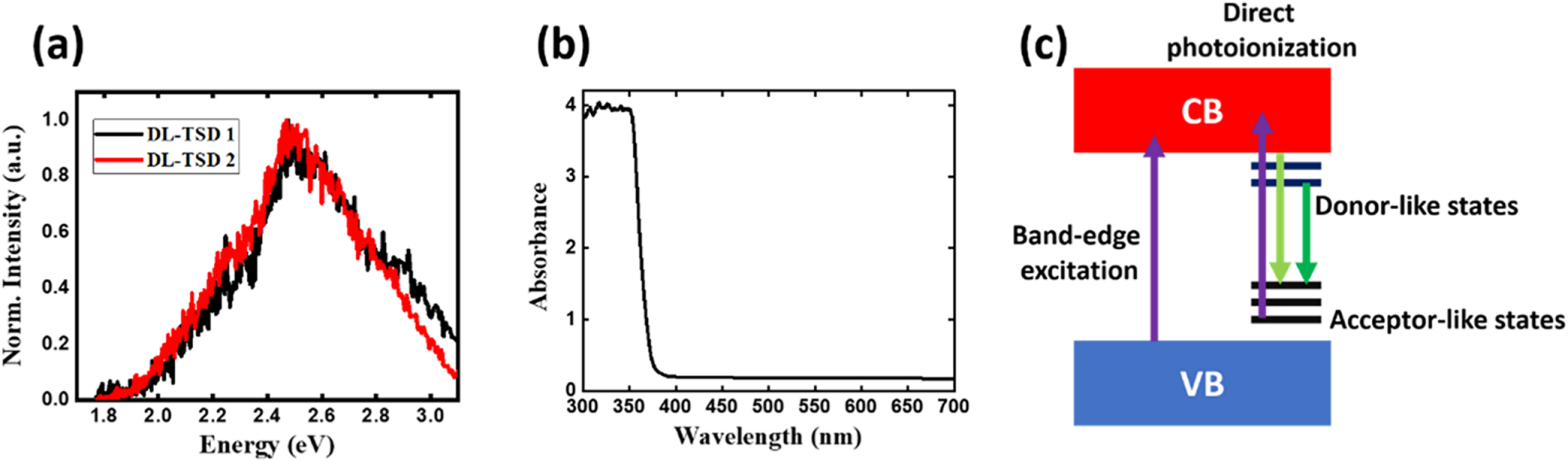
Probing the electronic properties of two specific TSDs with deep-level states. (a) Defect-PL spectra of the two deep-level TSDs in the HPSI-SiC substrate. (b) Corresponding absorption spectra showing only band-edge absorption, with no impurity-related features in the visible range. (c) Schematic of possible electronic transitions for deep-level TSDs in HPSI-SiC substrate.

In fact, the scenario is more complex for the TD-related deep-level emissions in HPSI-SiC. In addition to free-to-bound transitions, the DL-TDs can host both donor-like and acceptor-like deep-level states within the bandgap, as illustrated in Figure 6(c). This enables an intrinsic defect–induced DAP transitions, in which both donor-like and acceptor-like deep levels originate from the same dislocation-related deep states, rather than from extrinsic impurities. Consequently, two types of electronic transitions, including free-to-bound recombination and intrinsic DAP transitions, could occur concurrently at room temperature, giving rise to the observed ultrabroad TD-related emission spectra (also see Supporting Information Figure S3).

### 3.5 Relation between TDs and Leakage Current

S. I. Maximenko et al. employed electron beam–induced current (EBIC) measurements to examine the influence of TDs on carrier transport in SiC homoepitaxial layers.^20^ Their results showed that TSDs have a stronger impact on carrier diffusion lengths and exhibit enhanced recombination activity, arising from deep-level states associated with dislocation cores, compared with TEDs. However, the EBIC technique is not suitable for HPSI-SiC substrates because of their low intrinsic carrier concentration and high electrical resistivity. Moreover, such electrical characterization is typically applied at the device level and is not practical for materials-level inspection. Fujiwara et al. investigated the relationship between TD density and leakage current density in SiC diodes fabricated on heavily n-doped SiC substrates.^19^ Using reverse *I*–*V* measurements, device-level emission microscopy, and defect-selective etching, they demonstrated that the influence of TDs is device-type dependent, exerting a more pronounced effect on Schottky barrier diodes while having relatively minor impact on p–n junction diodes.

### 3.6 Electronic Properties of TDs in Doped SiC

Kelvin probe force microscopy (KPFM) has been employed to study the electronic properties of the etch pits of TDs (instead of dislocation lines themselves) in doped SiC. For example, Luo et al, examined the electronic and optical properties of the etch pits of etched TDs, using KPFM and micro-PL mapping.^36^ They show that both etch pits of TEDs and TSDs behave as donors in n-type SiC. In addition, their μ-PL measurements revealed broad PL spectra encompassing both near-band-edge emission and broadband defect-related emissions from TD etch pits, which closely resembled the emission characteristics of background regions. More recently, Tong et al. studied the electronic properties of TDs in p-type SiC and revealed the interaction between TDs and aluminum impurities.^45^

It should be noted that most previous studies using KPFM methods have focused on the electronic properties of dislocation etch pits (instead of dislocation lines), where the signals are determined by abundant surface states created by chemical etching, rather than directly probing the intrinsic electronic behavior of the dislocation lines themselves.^36,45^ Moreover, techniques such as KPFM and EBIC are not well-suited for HPSI-SiC substrates due to their high resistivity and surface charging effects. In contrast to previous studies, here we developed a purely optical technique applicable to HPSI-SiC substrate, capable of directly probing the inherent deep-level states associated with dislocation core lines. This contactless technique is particularly well-suited for the selective detection and characterization of device-relevant TDs in highly resistive HPSI-SiC substrates.

## 4 Conclusions

In conclusion, we have investigated the electronic properties of device-relevant DL-TDs in HPSI-SiC substrates using a purely optical method that circumvents the limitations of conventional electrical and band-edge PL-based approaches. By combining SEM and laser backscattering mapping of the etch-pit morphologies with confocal subsurface defect-PL measurements, we directly correlated dislocation types with their electronic activity. Our results show that only a small subset of TSDs exhibit optically active deep-level states, which manifest as broadband visible PL with a characteristic peak at ∼2.5 eV, highlighting their potential role as device-relevant defects. This work establishes a nondestructive, contactless optical technique for the selective detection of electrically active TDs and provides an insight into their electronic properties.

## Supplementary Material



## Data Availability

All data supporting the findings of this study are available within the article and its Supporting Information.

## Notes

Schematic of a threading dislocation in the partially etched configuration used for one-to-one structural–electronic correlation; surface and cross-sectional laser backscattering and defect-PL mapping of an optically inactive threading mixed dislocation; and power-dependent defect-PL measurement of deep-level threading dislocations (PDF)
