# Supplementary material for: Optical Access to the Electronic Nature of Device-Relevant Dislocations in High-Purity Semi-Insulating SiC
Source: ACS Omega. 2026 Jun 25;11(26):38814–22. doi: 10.1021/acsomega.6c02027 (PMC13347646; doi:10.1021/acsomega.6c02027)
Supplement: Supplementary file 1 [file ao6c02027_si_001.pdf]

Supporting Information

Optical Access to The Electronic Nature of Device-Relevant Dislocations in High-Purity Semi-Insulating SiC

Hsiu-Ming Hsu,[1,2,†] Irwan Saleh Kurniawan,[1,2,†] Russel Cruz Sevilla,[1,2,†] Ruth Jeane Soebroto,[1,2] Sheng-Hsiung Chang,[3] Troy Tsai,[4] Hsiu-Ying Huang,[1,2,*] Wen-Chung Li,[1,2,5,*] and Chi-Tsu Yuan,[1,2,*]

[1]Department of Physics, Chung Yuan Christian University, Taoyuan, Taiwan
[2]Research Center for Semiconductor Materials and Advanced Optics, Chung Yuan Christian University, Taoyuan, Taiwan
[3]Department of Optics and Photonics, National Central University, Taoyuan, Taiwan
[4]LEAP Semiconductor Corporation, Taoyuan, Taiwan
[5]WAFER WORKS, Taoyuan, Taiwan
[†]These authors contributed equally.
**ctyuan@cycu.edu.tw (C.-T.Y.)*

S1. TDs with a partially etched configuration

The TDs examined here comprise surface dislocation etch pits and their underlying dislocation lines, namely a partially etched TD configuration (Figure S1). This configuration enables structure–electronic correlation by sequentially mapping etch-pit morphology via laser backscattering and probing the electronic activity of the same subsurface dislocation lines via defect-PL mapping. A statistical one-to-one correlation analysis can reveal how specific dislocation types impact electronic activity through deep-level states.

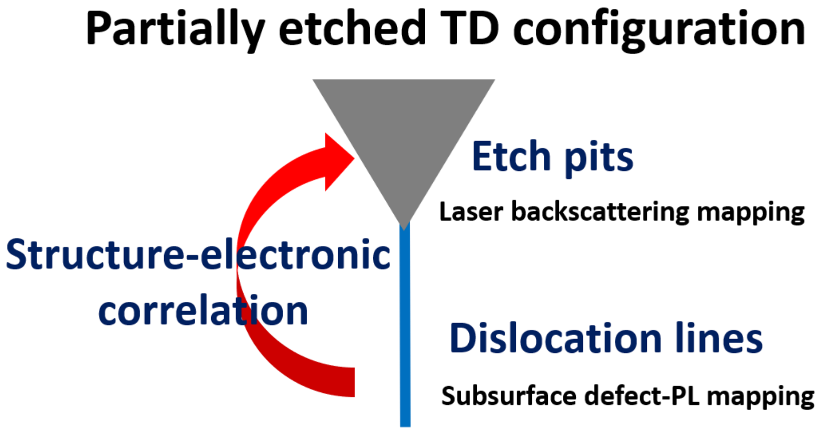

Figure S1. Schematic of a TD in the partially etched configuration, comprising a surface etch pit and the underlying dislocation line used for one-to-one structure–electronic correlation between etch-pit morphology (laser backscattering) and electronic activity (defect-PL).

S2. Optically inactive TMDs

Figure S2 presents a set of images, including laser backscattering surface and cross-sectional mappings of a representative TD. The observations reveal double hexagonal pits and curved depth profiles, characteristic of TMDs. In contrast, the depth-profile defect-PL image shows only bright emission from the etch pits, which originate from surface states, while no emission is detected along the underlying dislocation lines. This absence of deep-level mediated emission is further confirmed by subsurface defect-PL mapping. Therefore, these TDs can be identified as optically inactive TMDs, which are likely benign and irrelevant to device operation.

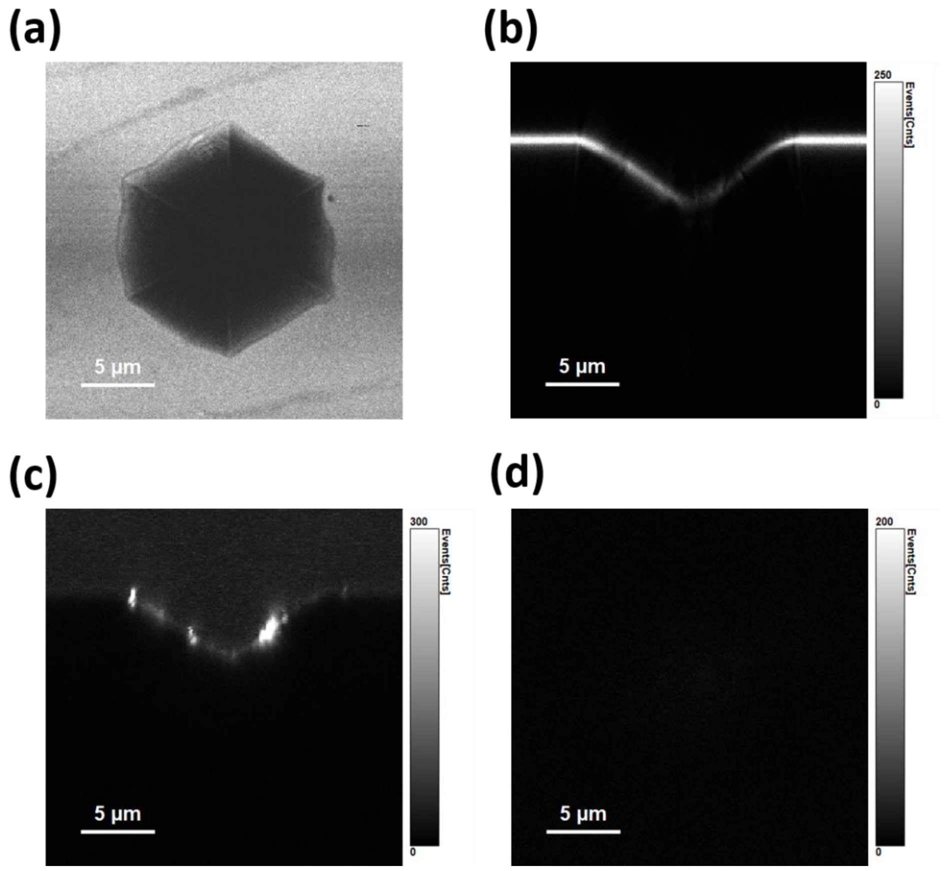

Figure S2. Optically inactive threading mixed dislocation (TMD). **(a)** Surface laser backscattering mapping of the etch pit, showing the double-hexagonal pit. **(b)** Cross-sectional laser backscattering mapping of the same etch pit, showing a curved depth

profile. **(c)** Cross-sectional defect-PL mapping, in which bright emission appears only at the etch pit and originates from surface states. **(d)** Subsurface defect-PL mapping, confirming the absence of deep-level emission along the underlying dislocation line.

S3. Power-dependent defect-PL measurement of DL-TDs

We have performed power-dependent PL measurements on the deep-level defect emission from DL-TDs (Figure S3). In SiC, defect-related emission generally involves two major recombination processes: free-electron-to-acceptor-bound-hole recombination (e–A emission) and donor–acceptor pair recombination (DAP emission). In the power-dependent PL measurements, two distinct emission bands were observed, and their relative intensity ratio changed with increasing excitation power. This power-dependent behavior arises because the e–A and DAP channels have different dependencies on the photoexcited carrier density: the e–A emission, which involves one free electron recombining with a bound hole, increases more readily with excitation power, whereas the DAP emission, which requires both donor-like and acceptor-like states to be simultaneously occupied, tends to saturate at higher excitation densities. These results indicate that at least two defect-related recombination channels are involved in the deep-level emission from DL-TDs, consistent with the coexistence of free-to-bound and intrinsic DAP transitions discussed in the main text.

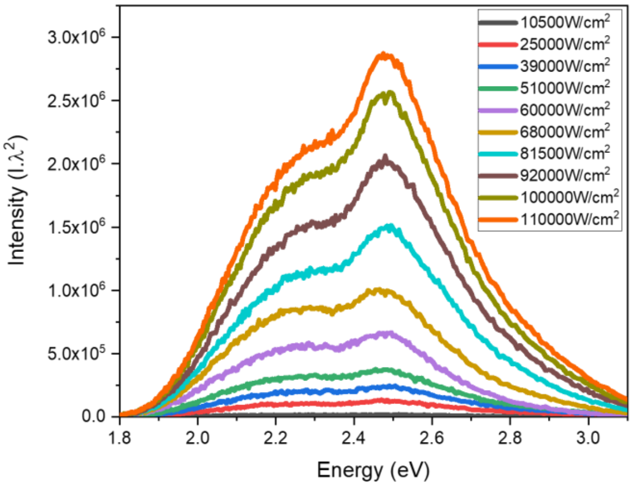

Figure S3. Power-dependent defect-PL of a deep-level threading dislocation (DL-TD). The relative intensity of the two emission bands shifts with excitation power, reflecting the distinct power dependences of the e–A (free-to-bound) and DAP recombination

channels, and confirming that at least two deep-level channels contribute to the DL-TD emission.
